# ER stress mediates Angiotensin II-augmented innate immunity memory and facilitates distinct susceptibilities of thoracic from abdominal aorta to aneurysm development

**DOI:** 10.3389/fimmu.2023.1268916

**Published:** 2023-09-04

**Authors:** Yifan Lu, Yu Sun, Fatma Saaoud, Ying Shao, Keman Xu, Xiaohua Jiang, Sheng Wu, Jun Yu, Nathaniel W. Snyder, Ling Yang, Xinghua Mindy Shi, Huaqing Zhao, Hong Wang, Xiaofeng Yang

**Affiliations:** ^1^ Centers of Cardiovascular Research, Lewis Katz School of Medicine at Temple University, Philadelphia, PA, United States; ^2^ Metabolic Disease Research and Thrombosis Research Center, Departments of Cardiovascular Sciences, Lewis Katz School of Medicine at Temple University, Philadelphia, PA, United States; ^3^ Medical Genetics and Molecular Biochemistry, Lewis Katz School of Medicine at Temple University, Philadelphia, PA, United States; ^4^ Department of Computer and Information Sciences, College of Science and Technology, Temple University, Philadelphia, PA, United States; ^5^ Biomedical Education and Data Sciences, Lewis Katz School of Medicine at Temple University, Philadelphia, PA, United States

**Keywords:** Ang II, ER stress, metabolic reprogramming, vascular inflammation, trained immunity

## Abstract

To determine the roles of endoplasmic reticulum (ER) stress and trained immunity, we performed transcriptome analyses on the thoracic aorta (TA) and abdominal aorta (AA) from the angiotensin II (Ang II)-HFD-ApoE-KO aneurysm model and made significant findings: 1) Ang II bypassed HFD-induced metabolic reprogramming and induced stronger inflammation in AA than in TA; 2) Ang II and HFD upregulated 890 genes in AA versus TA and induced cytokine signaling; 3) Ang II AA and TA upregulated 73 and 68 cytokines, scRNA-Seq identified markers of macrophages and immune cells, cell death regulators, respectively; transdifferentiation markers of neuron, glial, and squamous epithelial cells were upregulated by Ang II-AA and TA; and pyroptosis signaling with IL-1β and caspase-4 were more upregulated in Ang II-AA than in TA; 4) Six upregulated transcriptomes in patients with AAA, Ang II AA, Ang II TA, additional aneurysm models, PPE-AAA and BAPN-Ang II-AAA, were partially overlapped with 10 lists of new ER stress gene sets including 3 interaction protein lists of ER stress regulators ATF6, PERK, and IRE1, HPA ER localization genes, KEGG signal genes, XBP1 transcription targets, ATF4 (PERK) targets, ATF6 targets, thapsigargin ER stress genes, tunicamycin-ER stress genes, respectively; 5) Ang II-AA and TA upregulated ROS regulators, MitoCarta genes, trained immunity genes, and glycolysis genes; and 6) Gene KO transcriptomes indicated that ATF6 and PERK played more significant roles than IRE1 in promoting AAA and trained immunity whereas antioxidant NRF2 inhibited them. Our unprecedented ER-focused transcriptomic analyses have provided novel insights on the roles of ER as an immune organelle in sensing various DAMPs and initiating ER stress that triggers Ang II-accelerated trained immunity and differs susceptibilities of thoracic and abdominal aortas to diseases.

## Introduction

Aortic aneurysms are the second most common disease affecting the aorta after atherosclerosis, the 5th leading cause of death in individuals aged ≥ 55 years, and the 19th leading cause of death overall (1, 2), according to CDC Statistics (https://www.cdc.gov/injury/wisqars/LeadingCauses.html). Abdominal aortic aneurysms (AAA) are much more common than thoracic aortic aneurysms (TAA). AAA and TAA account for > 25000 deaths in the United States annually (3). Pathologically, AAAs are a localized dilatation of the aorta combined with vascular wall structure changes and the loss of vascular smooth muscle cells (VSMCs) in the aorta (4). Despite many advances in determining the roles of genetic risk factors (5), a significant question remains that which molecular mechanisms are underlying the inflammatory signal amplification for the localized dilatations that happened in specific areas of the aorta (6).

Trained immunity, or innate immune memory, is newly characterized metabolic reprogramming and epigenetic mechanisms underlying the persistent hyperresponsive phenotype that amplifies innate immune and inflammatory responses after brief stimulation (7–10). We recently reported that the aorta in pathologies is an immune organ (11) and innate immune cells, including endothelial cells (12, 13) and VSMCs (11, 14, 15) can develop exacerbated immunologic responses and long-term inflammatory phenotypes following brief exposure to endogenous or exogenous pathogen-associated molecular patterns (PAMPs)/danger-associated molecular patterns (DAMPs) (13, 16–19), which contribute to the pathophysiology of cardiovascular disease (CVD). However, an important question remained unknown, whether Angiotensin II (Ang II) and hyperlipidemia accelerate vascular inflammation and the progression of AAA *via* enhancing trained immunity in aortic vascular cells.

Although thoracic and abdominal aortic aneurysms share some common features, including the gross anatomic appearance, remodeling in the extracellular matrix, and loss of VSMCs, those two pathologies are distinct diseases (3). The theories of the embryologic origin of vascular cells raised in recent years, which illustrated the different phenotypes between the thoracic and abdominal aorta, may contribute to the progression of AAA (20). The embryologic origin of VSMCs in the thoracic aorta is from somites, whereas those in the abdominal aorta is from splanchnic mesoderm (21). In addition, the adventitia axon density (22), the vascular elastin density (23), and the phenotype of perivascular adipose tissue (24) were all different between the thoracic aorta and the abdominal aorta. However, an important question remains unknown whether the regional phenotypic differences of the aorta contribute to the development of AAA *via* different trained immunity and immune response patterns.

Our previous work demonstrated that the ER-dependent secretory pathway was modulated in AAA, which provides a microenvironment for immune cell activation and differentiation (11, 25). Targeting three ER transmembrane stress sensors, including protein kinase-like ER kinase (PERK, eukaryotic translation initiation factor 2 alpha kinase 3, EIF2AK3), inositol requiring kinase 1 (IRE1), and transcription factor-activating transcription factor 6 (ATF6) in AAA, is highly promising for potential therapeutics (26–30). However, the detailed molecular relationships between the three ER stress sensors in the pathologies of the thoracic and abdominal aorta and the establishment of trained immunity during the development of AAA remain poorly characterized.

In this study, to address the above-mentioned key knowledge gaps, we built an Ang II-induced AAA mouse model (31) and performed RNA sequencing (RNA-Seq) analysis (32) in the thoracic and abdominal aorta. We performed extensive transcription analysis to determine the roles of ER stress by using 13 ER stress-related gene lists, including one ER gene list from the Human Protein Atlas (HPA), one ER gene list from the KEGG database, three ER stress interactome gene lists from NCBI (PERK, IRE1, and ATF6), two ER stress inducers (Ca2+-ATPase inhibitor thapsigargin and glycosylation inhibitor tunicamycin) upregulated gene lists, three ER stress downstream transcription factors (XBP1, ATF4, and ATF6) target gene lists, and three ER stress regulator deficient (knockout, KO) datasets. In addition, we screened 79 groups of cell type marker gene lists (HPA database, https://www.proteinatlas.org/humanproteome/tissue+cell+type) from many different angles to illustrate the roles of ER stress in promoting trained immunity and contributing to the development of AAA and TAA. We made the following significant findings: 1) Angiotensin II stimulation bypasses high-fat diet (HFD)-induced metabolic reprogramming and induces strong inflammatory responses in the abdominal aorta but not in the thoracic aorta of atherogenic apolipoprotein E-knockout (ApoE-KO) mice; 2) most of the upregulated genes in the abdominal aorta and the thoracic aorta are not overlapped in response to angiotensin II (Ang II)-HFD stimulation, which indicates different areas of the aorta have different signatures in pathological conditions; 3) ATF6 and PERK ER stress pathways play more significant roles than the IRE1 pathway in promoting Ang II-ApoE-KO AAA gene upregulation as well as trained immunity gene upregulation. Taken together, our study provided a novel insight for understanding the role of ER stress in the development of AAA.

## Materials and methods

### Animal care

All animal experiments were performed per the Institutional Animal Care and Use Committee (IACUC) Guidelines and Authorization for the Use of Laboratory Animals and were approved by the IACUC of Temple University School of Medicine. ApoE-KO mice in a wild-type (WT, C57BL/6) background were obtained from the Jackson Laboratory (Bar Harbor, ME). ApoE-KO mice were weaned at three weeks of age and given surgery and a high-fat diet (HFD) at 9 –10 weeks.

### Angiotensin II-induced abdominal aortic aneurysm (AAA) model

Male ApoE-KO mice received saline or angiotensin II (Ang II, 1000 ng/kg/min) at 9–10 weeks old *via* mini-osmotic pumps (Alzet Model 2004, DURECT Corp.) for 28 days to induce AAA. During the 28 days, ApoE-KO mice were fed with HFD (0.2% (w/w) cholesterol and 20% (w/w) fat, Test Diet AIN-76A, Hubbard, OR). WT mice were fed with a normal chow diet (5% fat, Labdiet 5001).

### RNA sequencing (RNA-Seq) analysis

The whole aorta of mice was collected and separated into the abdominal and thoracic aortas by the diaphragm. GENEWIZ performed RNA extraction and RNA-seq analysis. Libraries containing Illumina adapter with TruSeq HT indexes were subsequently pooled and loaded into the Hiseq 2500. Single-end reads at 75 bp with 30 million reads per sample were generated for bioinformatics analysis. The RNA-seq row data has been deposited to NCBI-NIH (SUB13746947).

### Ingenuity pathway analysis and Metascape analysis

Ingenuity pathway analysis (IPA, Qiagen, Redwood City, CA) was used to characterize the clinical relevance and molecular and cellular functions related to the genes in our RNA-seq data. Differentially expressed genes were collected and uploaded to IPA for further analysis. Gene lists were uploaded to the Metascape website (https://metascape.org/gp/index.html#/main/step1).

## Results

### Angiotensin II stimulation bypassed HFD-induced metabolic reprogramming and induced stronger inflammatory responses in the abdominal aorta than those in the thoracic aorta of ApoE-KO mice

**Figure 1:**
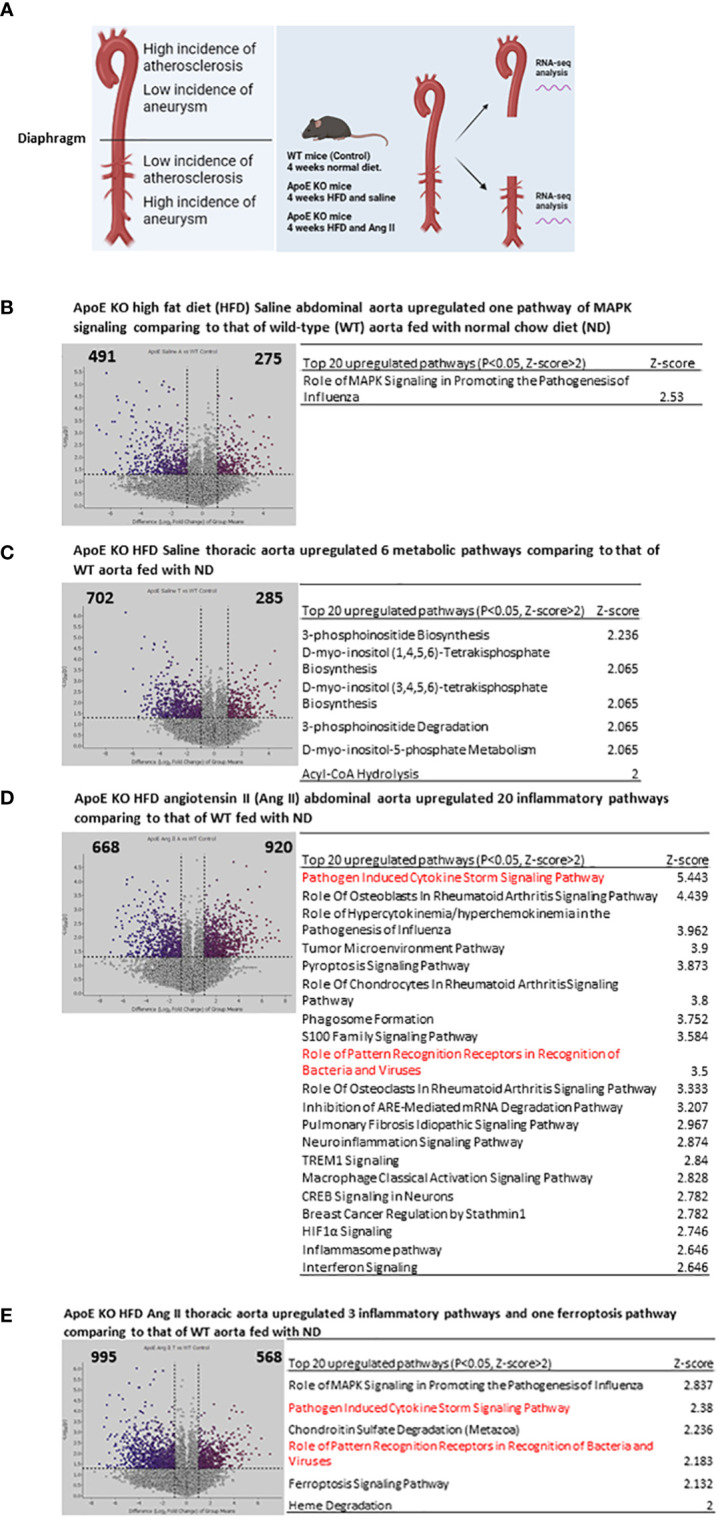
Four-week High fat diet feeding (HFD) induced 6 metabolic reprogramming pathways in ApoE KO thoracic aorta but induced only MAPK pathway ApoE KO abdominal aorta comparing to that of WT controls; and angiotensin II (AngII) and 4-week HFD (abdominal aortic aneurysm mouse model, AAA) induced 6 proinflammatory pathways in ApoE KO thoracic aorta but induced 20 proinflammatory pathways in ApoE KO abdominal aorta comparing to that of WT controls. **(A)** WT mice receive 4 weeks normal diet, ApoE KO mice receive 4 weeks HFD and Ang II, thoracic aorta and abdominal aorta were collected for RNA-seq. **(B)** Volcano plot indicates differentially expressed genes (DEG) in ApoE KO HFD Saline abdominal aorta vs WT normal diet (ND) condition, Top 20 upregulated pathways identified by IPA analysis of the DEG. **(C)** Volcano plot indicates differentially expressed genes (DEG) in ApoE KO HFD Saline thoracic aorta vs WT normal diet (ND) condition, Top 20 upregulated pathways identified by IPA analysis of the DEG. **(D)** Volcano plot indicates differentially expressed genes (DEG) in ApoE KO HFD Ang II abdominal aorta vs WT normal diet (ND) condition, Top 20 upregulated pathways identified by IPA analysis of the DEG. **(E)** Volcano plot indicates differentially expressed genes (DEG) in ApoE KO HFD Ang II thoracic aorta vs WT normal diet (ND) condition, Top 20 upregulated pathways identified by IPA analysis of the DEG. All the DEG cut off: P<0.05, LogFC>1 or <-1. IPA analysis cut off: P<0.05, Z-score>2.

The aortic arch and thoracic aorta develop higher incidences of atherosclerosis but a relatively lower incidence of Ang II-induced aortic aneurysm (33–37) than the abdominal aorta in the HFD-fed ApoE-KO plus Ang II infusion model (**Figure 1A**). However, the detailed transcriptomic mechanisms underlying the phenotypic differences remained poorly defined (5, 38–40). To determine the transcriptomic mechanisms, we performed RNA-Seq analysis to determine transcriptomic differences between the abdominal aorta and the thoracic aorta in AAA. As shown in **Figure 1B**, 275 genes were significantly upregulated in the abdominal aorta of ApoE-KO mice fed HFD plus saline (Ang II control) compared with WT mice fed ND. Ingenuity pathway analysis (IPA) indicated that one top pathway, “Role of mitogen-activated protein kinases (MAPK) Signaling in Promoting the Pathogenesis of Influenza”, was upregulated (*p* < 0.05, Z score > 2). In contrast, 285 genes were significantly upregulated in the thoracic aorta of ApoE-KO mice fed HFD plus saline (Ang II control) compared with WT mice fed ND. The IPA showed that six top pathways, including 3-phosphoinositide biosynthesis, D-myo-inositol-(1,4,5,6) tetrakisphosphate biosynthesis, D-myo-inositol-(3,4,5,6) tetrakisphosphate biosynthesis, 3-phosphoinositide degradation, D-myo-inositol-5-phosphate metabolism, and acyl-CoA hydrolysis, were upregulated in the thoracic aorta (**Figure 1C**), which indicated that the thoracic aorta more likely undergoes HFD-induced metabolic reprogramming than the abdominal aorta in ApoE-KO mice. Of note, recent reports showed that phosphatidylinositol (3,4,5)-trisphosphate (PI(3,4,5)P3) and PI(4,5)P2 are major PI 5-phosphatase substrates that show signaling properties related to AKT (protein kinase B), mitogen-activated protein kinase (MAPK)/extracellular signal regulated kinase (ERK) and cell survival (41); AKT, mammalian target of rapamycin (mTOR) or hypoxia inducible factor 1α (HIF1α)-mediated glycolysis functions metabolic basis for trained immunity (42); and immunity training requires MAPK signaling (43).

Surprisingly, we further found that 920 genes were significantly upregulated in the abdominal aorta of ApoE-KO mice fed HFD plus Ang II, which was 1.6 times more than that (568 upregulated genes) in the thoracic aorta (**Figures 1D, E**). The top 20 upregulated IPA pathways indicated that Ang II and HFD have much stronger synergies in inducing proinflammatory pathways in the abdominal aorta of ApoE-KO mice than the six top pathways in the thoracic aorta. The top 20 abdominal aorta pathways included pathogen-induced cytokine storm signaling, role of osteoblasts in rheumatoid arthritis signaling, role of hypercytokinemia and hyperchemokinemia, tumor microenvironment pathway, pyroptosis signaling, and other 15 proinflammatory pathways.

Taken together, these results showed that Ang II and HFD treatment significantly amplified the immune responses in the abdominal aorta without experiencing metabolic reprogramming. Since it has been reported that metabolic reprogramming is an essential step for the establishment of trained immunity (also termed innate immune memory) (8), hypercholesterolemia patients have a trained immunity phenotype, which accelerates vascular inflammation and atherosclerosis (44, 45). Although immune and inflammation mechanisms have been identified in the pathogenesis of abdominal aortic aneurysm (AAA) (46), our results have illustrated for the first time that Ang II, a well-documented risk factor for vascular disease, bypasses HFD-induced metabolic reprogramming and accelerates HFD-induced trained immunity specifically in the abdominal aorta but not in the thoracic aorta of ApoE-KO mice (47).

### Ang II and HFD upregulated 890 genes in abdominal versus thoracic aortas and induced more cytokine storm signaling, S100 family signaling, and phagosome formation in the abdominal aorta than in the thoracic aorta in ApoE-KO

**Figure 2:**
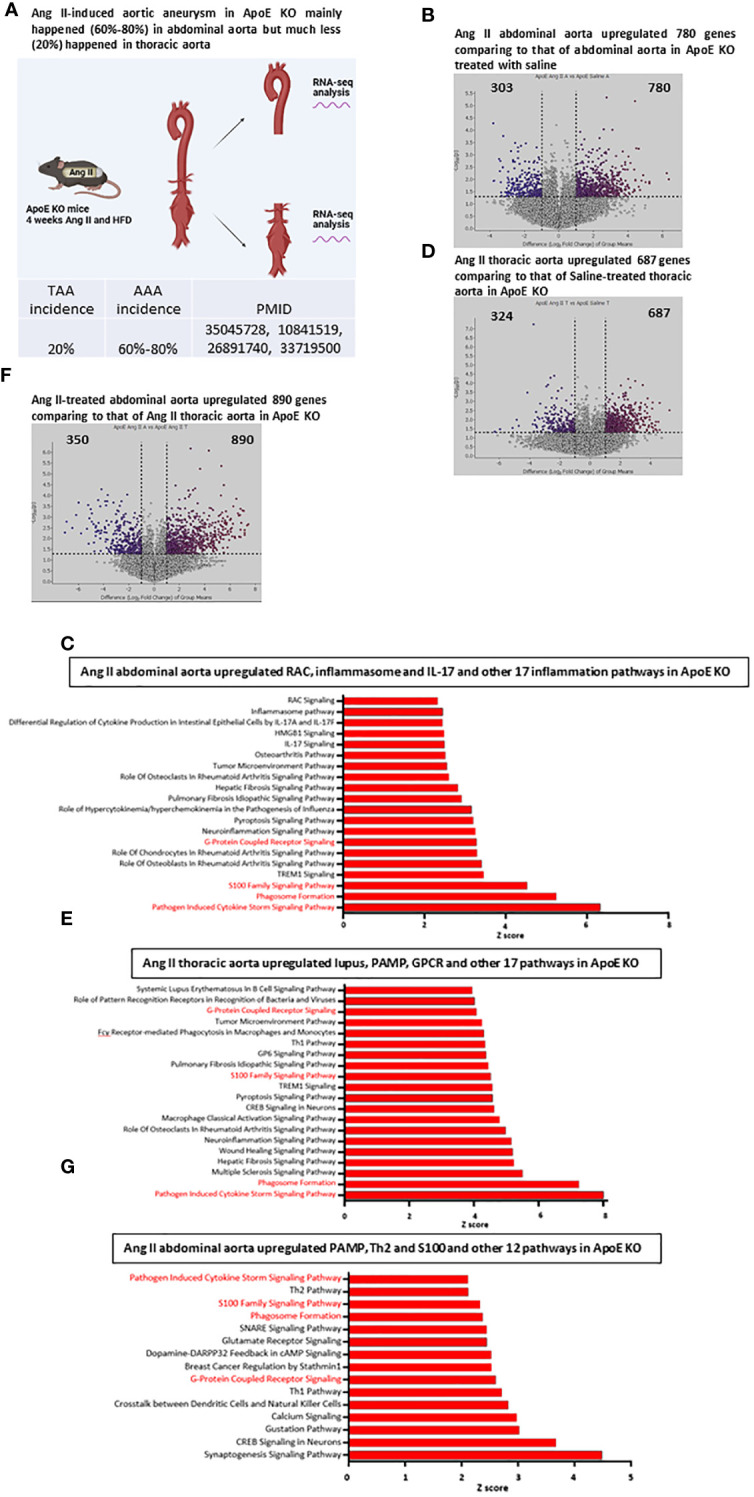
Ang II and HFD upregulate 780 genes in ApoE KO abdominal aorta, Ang II and HFD upregulate 687 genes in ApoE KO thoracic aorta; and Ang II and HFD in ApoE KO mice (AAA model) upregulated more cytokine storm signaling, S100 family signaling and phagosome formation in abdominal aorta than that in thoracic aorta. **(A)** Working model for study the difference between abdominal aorta and thoracic aorta by RNA-seq analysis in Ang II-induced abdominal aorta aneurysm mouse model. B and **(C)** Volcano plot indicates differentially expressed genes (DEG) in Ang II abdominal aorta vs Saline abdominal aorta condition **(B)**, Top 20 upregulated pathways **(C)** identified by IPA analysis of the DEG, red color indicates overlapped with other group. D and **(E)** Volcano plot indicates differentially expressed genes (DEG) in Ang II thoracic aorta vs Saline thoracic aorta condition **(D)**, Top 20 upregulated pathways identified **(E)** by IPA analysis of the DEG, red color indicates overlapped with other group. F and **(G)** Volcano plot indicates differentially expressed genes (DEG) in Ang II abdominal aorta vs Ang II thoracic aorta condition **(F)**, Top 20 upregulated pathways identified by IPA analysis of the DEG **(G)**, red color indicates overlapped with other group.

The Ang II-induced AAA is a well-documented mouse model and has been extensively characterized for more than 20 years (35). It has been reported that nearly 20% of aneurysms are developed in thoracic aortas and 60-80% of aneurysms are developed in abdominal aortas (**Figure 2A**). However, it remains poorly defined why aneurysm pathogenesis develops more in the abdominal aorta than in the thoracic aorta. We hypothesized that inflammatory transcriptomic responses to Ang II and HFD in the ApoE-KO abdominal aorta are stronger than those in the thoracic aorta. To test this hypothesis, we performed RNA-Seq analysis of the abdominal aorta and thoracic aorta in an Ang II-induced aneurysm model in comparison to their saline-treated ApoE-KO counterparts (**Figure 2A**). As shown in **Figure 2B**, 780 genes were upregulated in the Ang II abdominal aorta versus the saline abdominal aorta in HFD-fed ApoE-KO mice. The top upregulated pathways include the cytokine storm signaling pathway, phagosome formation, and the S100 family signaling pathway (**Figure 2C**). In the thoracic aorta of Ang II-stimulated HFD-fed ApoE-KO mice, 687 genes were upregulated by Ang II stimulation, which was lower than that in the abdominal aorta (**Figure 2D**). The 11 pathways among the top 20 pathways were shared between the Ang II abdominal aorta and the Ang II thoracic aorta. More importantly, the IPA showed that nine out of the top 20 pathways were identified specifically in the Ang II abdominal aorta, including RAC signaling, inflammasome signaling, differential cytokine production by IL-17A and IL-17F, HMGB1 signaling, IL-17 signaling, osteoarthritis pathway, role of hypercytokinemia and hyperchemokinemia, and role of chondrocytes and role of osteoblasts in rheumatoid arthritis signaling. The IPA also identified nine out of the top 20 pathways in the Ang II thoracic aorta, including systemic lupus in B cell signaling, role of pattern-recognition receptors, Fcγ receptor-mediated phagocytosis, Th1 pathway, GP6 signaling, CREB signaling, macrophage classical activation signaling, wound healing signaling, and multiple sclerosis signaling (**Figure 2E**). Then we compared the transcriptomic differences of the abdominal versus thoracic aorta in Ang II stimulation in ApoE-KO mice. We found that 890 genes were upregulated in the Ang II abdominal aorta versus the Ang II thoracic aorta (**Figures 2F, G**). Taken together, our results have demonstrated for the first time that: *1)* Ang II stimulation has significant tissue specificities in the abdominal aorta and the thoracic aorta; *2)* it has been reported that Ang II binds to the angiotensin II receptor (AT1R) to initiate the following ten functions (48), including vasoconstriction, sodium retention, aldosterone secretion, ADH secretion, increased sympathetic tone, cell proliferation, inflammation, fibrosis, angiogenesis, and thrombosis. However, by comparison to that reported (48), our new findings on Ang II functions and pathways in abdominal aorta versus thoracic aorta in HFD-fed ApoE-KO mice significantly improve our understanding of Ang II aortic section-specific functions from that in the figure of a recent review published in Circ. Res (3).; and *3)* interaction and synergies of Ang II and HFD in ApoE-KO background contribute significantly to pathological differences between abdominal versus thoracic aorta in developing different aortic diseases, including AAA and atherosclerosis (11, 49).

### Pyroptosis pathways with upregulation of IL-1β and caspase-4 were more significantly upregulated in Ang II abdominal aorta than in the thoracic aorta

**Figure 3:**
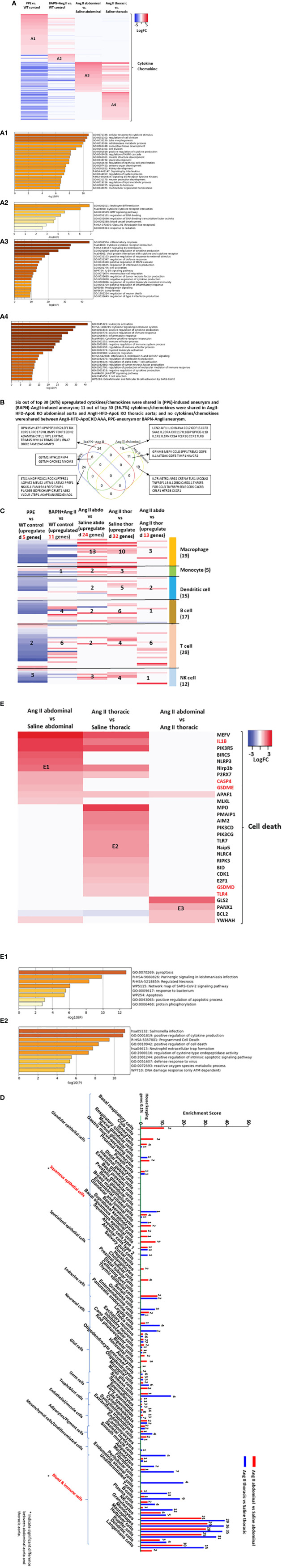
Ang II abdominal Aorta and Ang II thoracic aorta upregulated 73 and 68 cytokines, respectively, out of 1376 which are significantly different from the PPE-AAA upregulated 98 cytokines and BAPN-Ang II AAA upregulated 19 cytokines; and Six out of top 30 (20%) upregulated cytokines/chemokines were shared in (PPE)-induced aneurysm and (BAPN)-AngII-induced aneurysm; 11 out of top 30 (36.7%) cytokines/chemokines were shared in AngII-HFD-ApoE KO abdominal aorta and AngII-HFD-ApoE KO thoracic aorta; and no cytokines/chemokines were shared between AngII-HFD-ApoE KO AAA, PPE-aneurysm or BAPN-AngII aneurysm. **(A)** Heatmap of upregulated 1376 cytokine and chemokine genes (https://www.proteinatlas.org/search/cytokine) in different mouse model. **(B)** Venn diagram analysis of top 30 upregulated cytokine/chemokine in each group. **(C)** Heatmap of immune cell marker expression in different group. Immune cell marker gene list from PMID: 35549406. **(D)** Screen cell type marker list from human protein atlas, number indicates the number of upregulated cell type marker. **(E)** Heatmap of cell death regulator genes in Ang II abdominal vs Saline abdominal, Ang II thoracic vs Saline thoracic and Ang II abdominal vs Ang II thoracic condition. Upregulated genes in E1 and E2 were analysis by metascape pathway. Cut off: P<0.05, LogFC>1 or <-1.

We hypothesized that first, the abdominal aorta and thoracic aorta have differential cytokine and chemokine responses in response to Ang II stimulation in ApoE KO mice, and second, several AAA mouse models have differential cytokine and chemokine upregulation and increased cytokine signaling pathways. To examine those hypotheses, we screened the expression changes of a total of 1376 cytokine and chemokine genes from the HPA database, as we reported (11, 50, 51) in porcine pancreatic elastase (PPE)-induced AAA (52), β-aminopropionitrile monofumarate (BAPN, an irreversible inhibitor of lysyl oxidase) (53)-Ang II-induced AAA, and Ang II-induced AAA. As shown in **Figure 3A**, the abdominal aorta upregulated 73 cytokines and the thoracic aorta upregulated 68 cytokines, which did not overlap with each other. The 98 cytokines upregulated in PPE-AAA and the 19 cytokines upregulated in BAPN-Ang II AAA, which were different from those of Ang II-AAA. The cytokines and chemokines upregulated in BAPN-Ang II AAA were much less than those of PPE-AAA and Ang II. Further signaling pathway analysis indicated that the function of upregulated cytokines indicated that PPE-AAA (A1) included cellular response to cytokines, cell division, tube morphogenesis, nitrobenzene metabolism, connective tissue development, and 15 other pathways. BAPN-Ang II AAA (A2) promoted leukocyte differentiation, cytokine response, BMP signaling, transcription factor binding to DNA, and three other pathways. Ang II abdominal aorta (A3) upregulated the inflammatory response, cytokine signaling, interleukin signaling, and 17 other pathways, including cell death and fibrosis. Ang II thoracic aorta (A4) upregulated leukocyte activation, cytokine signaling in the immune system, positive regulation of cytokine production, positive regulation of immune response, and 16 other pathways, including TNF-α production, leukocyte migration, and ab T cell activation. The Venn diagram analysis of the top 30 upregulated cytokines and chemokines (**Figure 3B**) indicated that 6 out of 30 (20%) upregulated cytokines and chemokines were shared in PPE-induced AAA and BAPN-Ang II-induced AAA; 11 out of 30 (36.7%) cytokines and chemokines were shared in Ang II-AAA abdominal aorta and Ang II-AAA thoracic aorta; and no cytokines and chemokines were shared between Ang II-AAA, PPE-AAA, or BAPN-Ang II AAA.

Based on the results of cytokines and chemokines upregulated in the aortic transcriptomes of three AAA mouse models, we screened the transcriptomic changes of 299 immune cell markers (54), including macrophages, monocytes, dendritic cells, B cells, T cells, and NK cells, which can partially indicate immune cell infiltration in the aorta. As shown in **Figure 3C**, the Ang II abdominal aorta upregulates 13 macrophage genes, the highest number of macrophage genes among five datasets. The Ang II thoracic aorta upregulates 10 macrophage genes and 6 B cell marker genes. These results indicated that macrophages are the major cell type recruited to the abdominal aorta, and macrophages and B cells are the major cell types recruited to the thoracic aorta, which contribute to inflammation in Ang II-induced AAA. Our data demonstrated that Ang II-induced AAA has significantly higher numbers of immune cell recruitments than PPE AAA and BAPN-Ang II-AAA, which were well correlated with those reported (3).

An important question remains whether cell transdifferentiation takes place in the pathogenesis of AAA (55). To address this question, we examined the transcriptomic changes of the cell markers (8065 cell markers) of the whole 79 cell types in **Figure 3D**. Interestingly, we also found that the markers of neuron cells and glial cells were upregulated by Ang II stimulation, indicating the role of the newly established nervous system in the pathogenesis of AAA. In addition, comparing the abdominal aorta with the thoracic aorta, we found that the markers of squamous epithelial cells were only significantly upregulated in the abdominal aorta but not in the thoracic aorta.

Based on these results of pyroptosis-released IL-1β (11, 19) upregulated in Ang II-induced abdominal aorta in **Figure 3B**, we hypothesized that the increased immune cell infiltration and cytokine release in Ang II-induced AAA would further promote cell death. As shown in **Figure 3E**, AAA aortas upregulated cell death regulators were significantly different. Among them, caspase-4, gasdermin E (GSDME), and IL1β were significantly upregulated in the abdominal aorta but not in the thoracic aorta in Ang II-induced AAA, which indicates the non-canonical inflammasome-pyroptosis pathway may contribute to cell death in the abdominal aorta. The pathway analysis of upregulated cell death genes in each group indicated that seven pathways, such as pyroptosis, purinergic signaling, necrosis, SARS-CoV2 signaling, response to bacteria, apoptosis, and protein phosphorylation pathways were upregulated in the abdominal aorta, and in contrast, nine pathways were upregulated in the thoracic aorta.

### The upregulated genes in the aortas of patients with abdominal aortic aneurysm, Ang II abdominal and thoracic aortas partially overlapped with the interactomes of ER stress regulators ATF6, PERK, and IRE1, HPA-ER localization protein genes, and KEGG ER stress signaling genes

**Figure 4:**
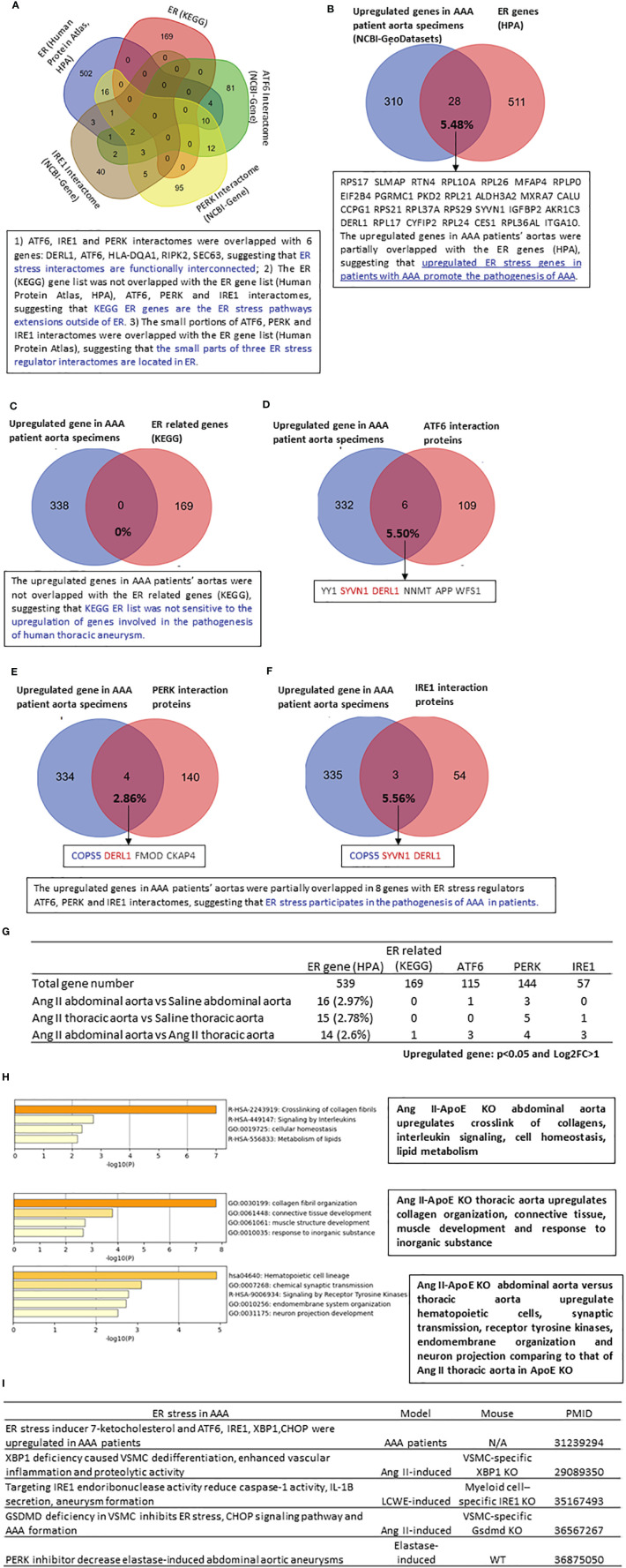
The upregulated genes in AAA patients’ aortas are partially overlapped with ER stress regulators ATF6, PERK and IRE1 interactomes, suggesting that ER stress participates in the pathogenesis of AAA; and the ER genes (HPA) and ER stress regulators interactomes are upregulated more (11 genes) in Ang II-HFD treated ApoE KO abdominal aorta than that (6 genes) in Ang II-HFD treated ApoE KO thoracic aorta. **(A)** Venn diagram of 539 ER gene from human protein atlas (https://www.proteinatlas.org/search/subcell_location%3AEndoplasmic+reticulum) 169 ER related gene from KEGG (https://www.genome.jp/dbgetbin/wwwbget?pathway:hsa04141 115 ATF6 interaction protein (https://www.ncbi.nlm.nih.gov/gene/22926) 144 PERK interaction protein (https://www.ncbi.nlm.nih.gov/gene/9451) 57 IRE1 interaction protein (https://www.ncbi.nlm.nih.gov/gene/2081). **(B–F)** Venn diagram of upregulated genes in AAA patients aortic specimens (NCBI-GeoDatasets-GSE57691) with Mitocarta genes (4.70%), ER genes (5.48%), ER related genes (0%), ATF6 interaction proteins (5.50%), PERK interaction proteins (2.86%) and IRE1 interaction proteins (5.56%). **(G)** The number of upregulated mitocarta and ER stress genes in each group. **(H)** Metascape analysis of upregulated ER genes in each group. (I) The recent study of ER stress in AAA. All of the differently expressed genes for this analysis cut off: P<0.05 and LogFC>1.

ER, the largest cellular organelle, is responsible for secretory and transmembrane protein folding. Disruption in the ER protein-folding system leads to ER stress, which contributes to the progression of CVD, including atherosclerosis and hypertension (56). In addition, upregulated ER stress has been reported in patients with AAA and gene-mutant mouse models of AAA (**Figure 4I**). However, the transcriptomic changes associated with ER stress in AAA have been poorly characterized previously. We hypothesized that three ER stress pathways (57), including ATF6, PERK, and IRE1, contribute to the progression of AAA. We found that only small portions of the ATF6 (125 interaction proteins), PERK (144 interaction proteins), and IRE1 (57 interaction proteins) interactomes overlapped with the 538 HPA-ER localization protein gene list, suggesting that the small parts of the three ER stress regulator interactomes are localized in the ER (**Figure 4A**). The ATF6, IRE1, and PERK interactomes overlapped with six genes: DERL1, ATF6, HLA-DQA1, RIPK2, and SEC63, suggesting that three ER stress regulator interactomes are functionally interconnected. Then we did Venn diagram analysis to determine whether those HPA-ER genes/ER stress interactomes were upregulated in human thoracic aneurysm samples. We found that 5.48% of ER genes (28 genes) were upregulated in human AAA, suggesting the potential roles of ER genes in promoting AAA (**Figure 4B**). However, 169 KEGG-ER-related signaling genes from the KEGG database (https://www.genome.jp/kegg/pathway.html) were not sensitive to the upregulation of genes involved in the pathogenesis of human thoracic aneurysms (**Figure 4C**). One of the explanations for why the KEGG pathway genes were not sensitive to being modulated is that the KEGG pathway focuses on post-translational signaling such as phosphorylation and de-phosphorylation due to the history of fields. As shown in **Figures 4D–F**, the upregulated genes in thoracic aneurysm patients’ aortas were partially overlapped in 8 genes with ER stress regulators ATF6, PERK, and IRE1 interactomes, suggesting that ER stress participates in the pathogenesis of thoracic aneurysm in patients. Similar to human AAA, 20 HPA ER genes, KEGG-ER stress regulators, and ER stress regulator interactomes were upregulated in Ang II-HFD treated ApoE-KO abdominal aorta, and 21 ER genes and ER stress regulators and interactome genes were upregulated in Ang II-HFD-treated ApoE-KO thoracic aorta (**Figure 4G**).

In a detailed analysis of the functions of those upregulated ER genes, we found that in AAA, the abdominal versus thoracic aorta upregulated hematopoietic cells, synaptic transmission, receptor tyrosine kinases, endomembrane organization, and neuron projection (**Figure 4H**). Taken together, the results have demonstrated that three ER stress pathways, ATF6, PERK, and IRE1, participate in the pathogenesis of human aortic aneurysms and mouse Ang II abdominal aortic and thoracic aneurysms. Our findings are well correlated with previous reports in patients with AAA (26), and AAA mouse models, including VSMC-specific IRE1 downstream transcription factor X-box binding protein 1 (XBP1) (58)-KO mice (27), myeloid-specific IRE1-KO mice (28), VSMC-specific gasdermin D (GSDMD)-KO mice (59), and elastase-induced AAA treated with a PERK inhibitor (29) (**Figure 4I**).

### Aorta from Ang II induced-abdominal AA upregulated 66 XBP1 targets, 194 ATF4-PERK targets, and 38 ATF6 targets; and the aorta from Ang II induced-thoracic AA upregulated 49 XBP1 targets, 259 ATF4-PERK targets, and 19 ATF6 targets

**Figure 5:**
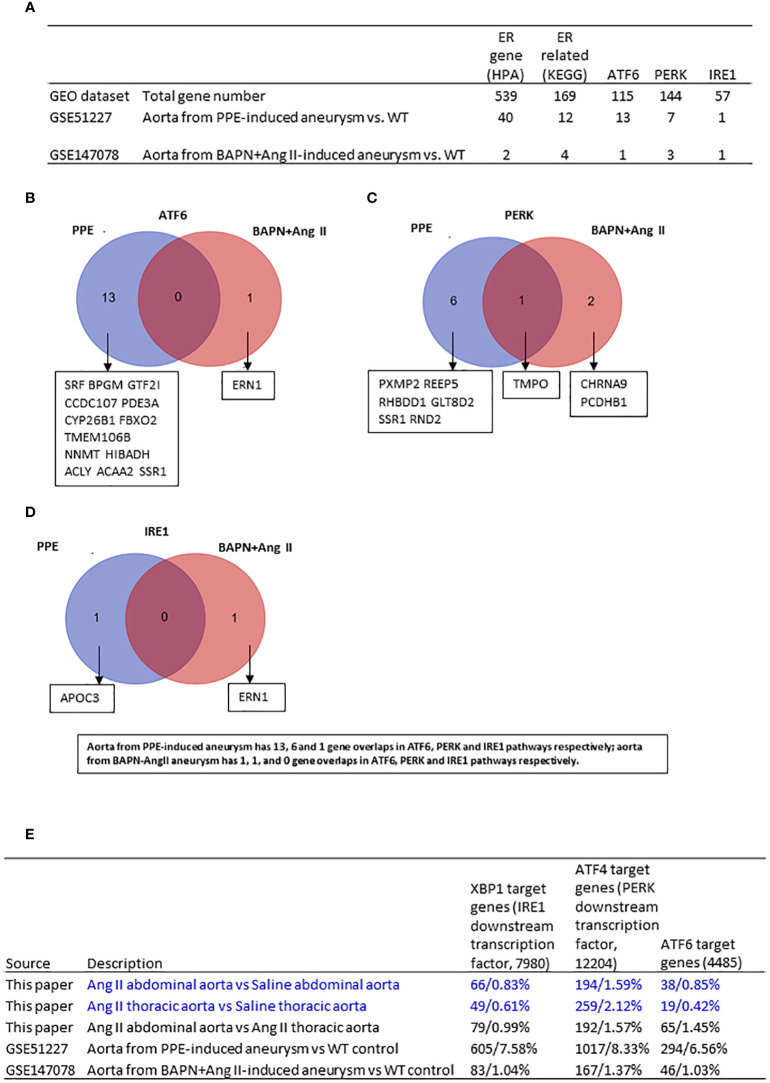
Ang II-ApoE KO abdominal aorta upregulated more XBP1—IRE1 target genes, ATF6 target genes but less ATF4-PERK target genes than of Ang II-ApoE KO thoracic aorta; and ER stress genes are upregulated in porcine pancreatic elastase (PPE)-induced AAA model more than that beta-aminopropionitrile BAPN+Ang II-induced AAA mouse model. **(A)** The number of upregulated ER stress genes in each group. **(B-D)** Venn diagram analysis of upregulated ATF6, PERK, IRE1 interactome in different mouse model. **(E)** Screen three transcription factors which are downstream of three ER stress pathways in each dataset. XBP1, ATF4 and ATF6 target genes from ChIP-Atlas. Cut off: P<0.05, LogFC>1 or <-1.

To fully address the roles of ER stress in the pathogenesis of AAA, we further screened the expression changes of ER stress genes in other two different AAA mouse models. As shown in **Figure 5A**, the 73 HPA ER localization protein genes, KEGG ER stress signaling genes, and ER stress regulator interactome genes were upregulated in PPE-induced aneurysms, and the 11 HPA ER genes, KEGG ER stress genes, and ER stress regulator interactome genes were upregulated in BAPN-Ang II-induced aneurysms. The Venn diagram analysis of upregulated ER stress interactomes from the two AAA models indicated that only one interactome gene from the PERK pathway was overlapped, suggesting that ATF6, PERK, and IRE1 ER stress pathways play different roles in PPE-AAA, BAPN-Ang II AAA, Ang II abdominal AAA, and Ang II thoracic AAA (**Figures 5B–D**).

The KEGG ER stress signaling proteins and the three ER stress regulators ATF6, PERK, and IRE1 interactomes carry out the functions in post-translational manners, and HPA ER proteins are all localized in the ER, which may not always play roles in ER stress functions. Therefore, we hypothesized that upregulation of ER stress downstream transcription factors (TFs) such as ATF6, PERK downstream ATF4, and IRE1 downstream XBP1 target genes in AAA can better indicate the roles of three ER stress signaling pathways in the pathogenesis of AAA. We determined the expression changes of 7980 IRE1 downstream XBP1 target genes collected from a comprehensive database of potential transcription factor target genes (ChIP-Atlas | Target genes (dbcls.jp)), 12204 PERK downstream TF ATF4 target genes, and 4485 ATF6 target genes in AAA transcriptomes. We found that: 1) aorta from PPE-aortic aneurysm upregulated 605 XBP1 target genes, 1017 ATF4–PERK target genes, and 294 ATF6 target genes, respectively; 2) aorta from BAPN-Ang II aortic aneurysm upregulated 83 XBP1 target genes, 167 ATF4–PERK target genes, and 46 ATF6 target genes, respectively; 3) abdominal aorta from Ang II-induced AAA upregulated 66 XBP1 target genes, 194 ATF4–PERK target genes, and 38 ATF6 target genes, respectively; 4) thoracic aorta from Ang II-induced AAA upregulated 49 XBP1 target genes, 259 ATF4–PERK target genes, and 19 ATF6 target genes, respectively; and 5) abdominal aorta versus thoracic aorta from Ang II-induced AAA upregulated 79 XBP1 target genes, 192 ATF4–PERK target genes, and 65 ATF6 target genes, respectively. The results demonstrated that i) among three aneurysm models, the numbers of upregulated genes in three ER stress TF potential target genes in the aorta from BAPN-Ang II AAA were at a similar level to that of Ang II AAA; and ii) the numbers of upregulated genes in three ER stress TF potential target genes in Ang II-ApoE-KO abdominal aorta upregulated more XBP1—IRE1 target genes and ATF6 target genes but less ATF4-PERK target genes than that of Ang II-ApoE-KO thoracic aorta (**Figure 5E**).

### 35 genes from thapsigargin-induced ER stress (TIES genes) were upregulated in Ang II abdominal AA; 28 TIES genes were upregulated in Ang II thoracic AA; 52 genes from tunicamycin-induced ER stress (TUES genes) were upregulated in Ang II abdominal AA; and 42 TUES genes were upregulated in Ang II thoracic AA, which all contributed to inflammatory response, cytokine production, innate immune response and immunity

**Figure 6:**
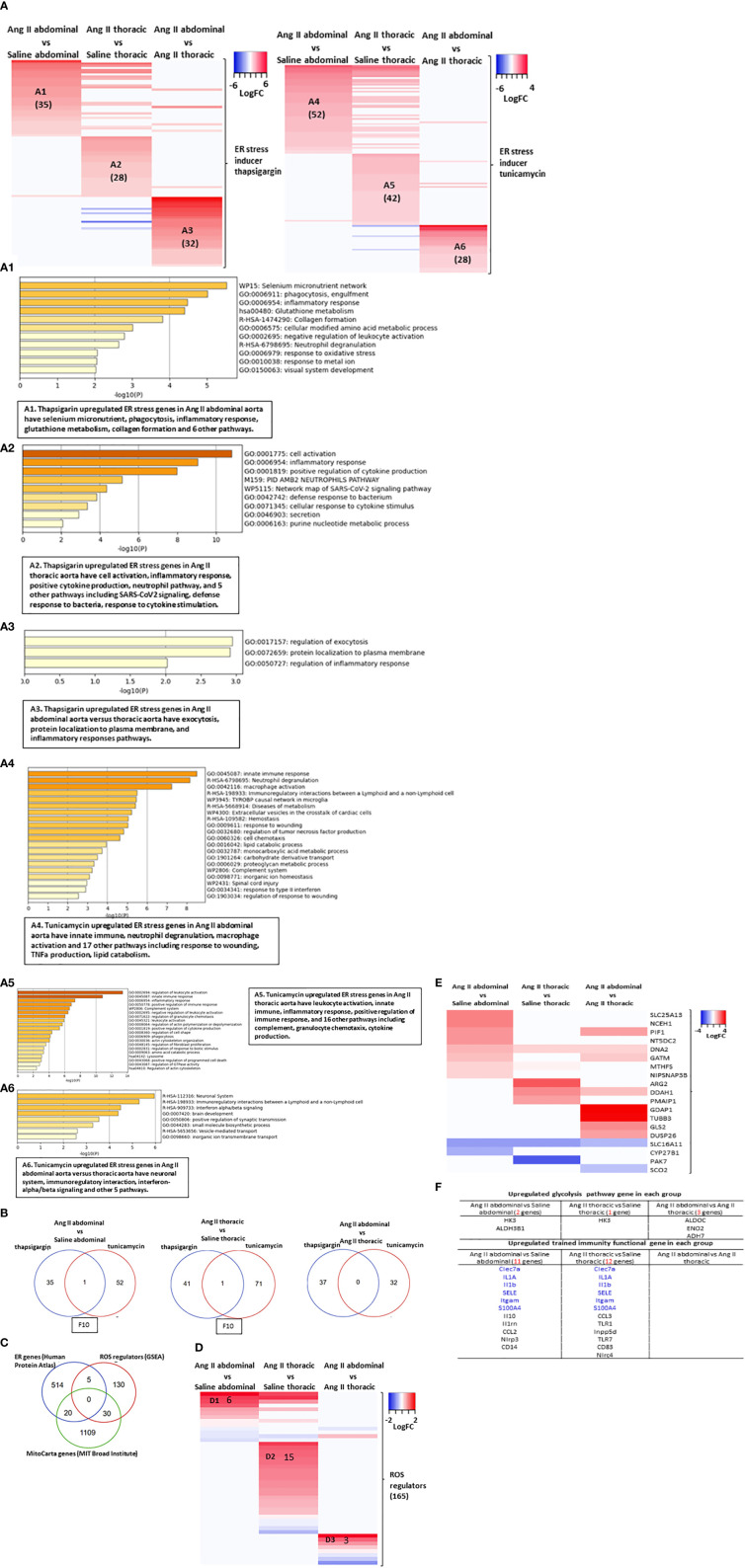
Abdominal aorta and thoracic aorta are different organ in response to ER stress, ROS and cell death. **(A)** Heatmap of ER stress inducer thapsigargin (TG, sarco/endoplasmic reticulum Ca ATPase inhibitor) upregulated 1308 genes from (GSE200626) and ER stress inducer tunicamycin (UDP-HexNAc: polyprenol-P HexNAc-1-P enzyme family antibiotic inhibitor) upregulated 1816 genes from (GSE167299) in Ang II abdominal vs Saline abdominal, Ang II thoracic vs Saline thoracic and Ang II abdominal vs Ang II thoracic condition. **(B)** Heatmap of three ER stress pathway interaction proteins in Ang II abdominal vs Saline abdominal, Ang II thoracic vs Saline thoracic and Ang II abdominal vs Ang II thoracic condition. **(C)** Venn diagram of ER gene, 165 ROS regulator from GSEA and mitoCarta genes. **(D)** Heatmap of 165 ROS regulator from GSEA in Ang II abdominal vs Saline abdominal, Ang II thoracic vs Saline thoracic and Ang II abdominal vs Ang II thoracic condition. **(E)** Heatmap of mitoCarta genes in Ang II abdominal vs Saline abdominal, Ang II thoracic vs Saline thoracic and Ang II abdominal vs Ang II thoracic condition. **(F)** Screen trained immunity pathway gene and trained immunity functional gene in each group.

Although some downstream regulators of ER stress, including XBP1 (27), IRE1 (28), or PERK (29), have been identified as potential targets for AAA, the detailed molecular mechanisms of experimentally verified ER stress genes in specific areas of the aorta (abdominal or thoracic aorta) are poorly documented. We hypothesized that ER stress-induced genes partially overlapped with upregulated genes in Ang II abdominal AA and thoracic AA. We examined 1039 thapsigargin-upregulated ER stress genes in the NCBI-Geodatasets database (https://www.ncbi.nlm.nih.gov/gds/?term=GSE200626) and 1817 tunicamycin-upregulated ER stress genes (GSE167299) in our Ang II-induced AAA RNA-Seq data. We found that the upregulated ER stress genes in the abdominal aorta and thoracic aorta were different, suggesting the abdominal aorta and thoracic aorta have different ER stress pathways (**Figure 6A**). The 35 genes from thapsigargin-induced ER stress (TIES genes) were upregulated in Ang II abdominal AA (A1), which promoted selenium microenvironment, phagocytosis, inflammatory response, glutathione metabolism, collagen formation, cellular modified amino acid metabolic process, and five other pathways; the 28 TIES genes were upregulated in Ang II thoracic AA (A2), which promoted cell activation, inflammatory response, positive regulation of cytokine production, neutrophil pathway, and five other pathways; the 32 TIES genes were upregulated in Ang II abdominal AA versus thoracic AA (A3), which promoted regulation of exocytosis, protein localization to the plasma membrane, and regulation of inflammatory response. In comparison, the 52 genes from tunicamycin-induced ER stress (TUES genes) were upregulated in Ang II abdominal AA (A4), which promoted innate immune response, neutrophil degranulation, macrophage activation, immunoregulatory interaction, Tyrobp casual network of microglia, diseases of metabolism, and 14 other pathways; the 42 TUES genes were upregulated in Ang II thoracic AA (A5), which promoted regulation of leukocyte activation, innate immune response, inflammatory response, positive regulation of immune response, and 16 other pathways; the 28 TUES genes were upregulated in the Ang II abdominal AA versus thoracic AA (A6), which promoted neuronal system, immunoregulatory interactions, interferon alpha/beta signaling, brain development positive regulation of synaptic transmission, and three other pathways. The Venn diagram showed that (**Figure 6B**): TIES genes have one gene overlapped with TUES genes in Ang II abdominal Aorta upregulated genes and in Ang II thoracic Aorta upregulated genes; TIES genes have no genes overlapped with TUES genes in Ang II abdominal Aorta upregulated genes versus Ang II thoracic aorta upregulated genes. These results have demonstrated the diversity of aortic regions in response to ER stress.

### ROS regulators had 5 genes overlapped with HPA-ER localization genes and 30 genes overlapped with MitoCarta genes; Ang II-ApoE-KO AAA upregulated 11 genes from MitoCarta genes (0.95%) and 31 HPA-ER localization genes (5.75%); and Ang II + HFD upregulated a list of 17 trained immunity genes but two glycolysis genes without inducing significant metabolic reprogramming

Previous reports showed that ROS plays significant roles in promoting the pathogenesis of aortic aneurysms (60–62) *via* enhancing inflammatory cell infiltration, smooth muscle cell migration, and apoptosis in AAAs (63). We also reported that ROS in the ER, mitochondria, and other organelles (64) are interconnected in sensing metabolic stress (65). We hypothesized that ROS in the ER, mitochondria, and other organelles mediate ER stress signaling in AAA. As shown in **Figure 6C**, HPA-ER localization protein genes shared 5 genes with ROS regulators (GSEA), including TLR6, PID1, PRKCD, AKR1C3, and SELENOS, suggesting that ER is an important organelle in regulating ROS. In addition, HPA-ER genes also shared 20 genes with the nuclear genome-encoded mitochondrion localization protein genes (MitoCarta genes, MIT-Broad Institute, https://www.broadinstitute.org/mitocarta/mitocarta30-inventory-mammalian-mitochondrial-proteins-and-pathways), suggesting that ER and mitochondria are functionally interconnected through ER-mitochondrial membrane tethering (64). Moreover, ROS regulators had 30 genes that overlapped with the MitoCarta genes, suggesting that mitochondria are a significant organelle in regulating ROS, as we and others reported (65). Following this idea, we found the upregulated ROS regulators in three groups of aortic upregulated genes have minimal overlaps. These results have demonstrated that ROS signals contribute to the pathogenesis of Ang II-ApoE-KO AAA (**Figure 6D**). Similar, the upregulated MitoCarta genes in three groups of aortic upregulated genes have minimal overlaps (**Figure 6E**).

Because metabolic reprogramming is an essential step for trained immunity (8) and ER and mitochondria play significant roles in metabolic processes (65). In **Figure 1**, we found that Ang II bypassed signaling pathways in metabolic reprogramming in ApoE-KO mice with HFD. To further determine whether Ang II + HFD stimulation has the ability to amplify immune response without metabolic reprogramming, we screened 102 metabolic enzymes involved in the three trained immunity metabolic pathways, including the glycolysis pathway, mevalonate pathway, and acetyl-CoA pathway (16) and also 101 trained immunity genes collected in the Trained Immunity Database (https://academic.oup.com/database/article/doi/10.1093/database/baab041/6318070). Surprisingly, we found only 2 glycolysis genes were upregulated in Ang II + HFD stimulation, suggesting that Ang II and HFD-feeding bypassed the metabolic reprogramming for AAA in ApoE-KO mice (**Figure 6F**). Without upregulating enzyme genes involved in metabolic reprogramming, we still found that 11 trained immunity functional genes were upregulated in the abdominal aorta and 12 genes were upregulated in the thoracic aorta. Taken together, these results have demonstrated that 1) ROS regulators had 5 genes overlapped with ER localization protein genes (HPA); and ROS regulators had 30 genes overlapped with the MitoCarta genes, suggesting that mitochondria may play more roles in regulating ROS; 2) Ang II-induced AAA in ApoE-KO upregulates 11 MitoCarta genes (0.95%) and 31 HPA-ER localization protein genes (5.75%) (**Figure 4G**), suggesting that ER stress contributes more than mitochondrial stress in promoting the pathogenesis of Ang II-induced AAA in ApoE-KO; and 3) Ang II + HFD upregulate a list of 11 trained immunity genes but only upregulate two glycolysis enzyme genes, suggesting that Ang II + HFD have the potential ability to induce trained immunity while inducing significant metabolic reprogramming.

### ATF6 and PERK ER stress pathways played more significant roles than the IRE1 pathway in promoting Ang II-induced AAA gene upregulation in ApoE-KO as well as trained immunity gene upregulation; Nrf2 inhibited, but NOX2 and SET7 promoted Ang II-induced AAA in ApoE-KO transcriptome upregulation and trained immunity gene upregulation

**Figure 7:**
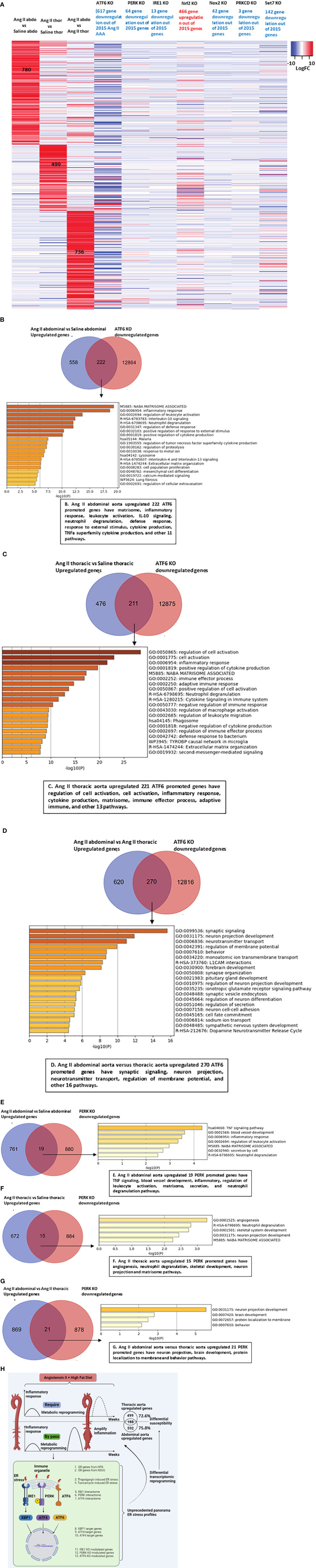
ATF6 deficiency inhibits the vascular inflammation in both abdominal aorta and thoracic aorta. **(A)** Heatmap of total upregulated genes (Ang IIabdominal vs Saline abdominal, Ang II thoracic vs Saline thoracic and Ang II abdominal vs Ang II thoracic) in ATF6 KO from GSE49646, PERK KO from GSE29929, IRE1 KO from GSE31683, Nrf2 KO from GSE7810, Nox2 KO from GSE100671, PRKCD SiRNA from GSE55503 and SET7 KO from GSE53038. **(B–D)** Venn diagram of upregulated genes in Ang II abdominal aorta, thoracic aorta and downregulated genes from ATF6 KO from GSE49646, overlapped genes were analyzed by metascape. **(E–G)** Venn diagram of upregulated genes in Ang II abdominal aorta, thoracic aorta and downregulated genes from PERK KO GSE29929, overlapped genes were analyzed by metascape. **(H)** Working model.

Our results so far have demonstrated that ER stress, ROS, and trained immunity may contribute to the pathogenesis of Ang II-induced AAA and TAA. We hypothesized that ER stress, ROS, and trained immunity have causative effects in promoting the pathogenesis of Ang II-induced AAA and TAA. To quantify the roles of three ER stress regulators, ATF6, PERK, and IRE1, we screened the expression changes of Ang II-HFD upregulated genes in the abdominal aorta, Ang II-HFD upregulated genes in the thoracic aorta, Ang II-HFD upregulated genes in the abdominal versus thoracic aorta in the ATF6-KO, PERK-KO, and IRE1-KO datasets. As shown in **Figure 7A**, the results showed that 617 genes, 64 genes, and 13 genes were downregulated in the ATF6-KO dataset, the PERK-KO dataset, and the IRE1-KO dataset, respectively, out of 2015 genes upregulated in the Ang II abdominal aorta (780 genes), the Ang II thoracic aorta (499 genes), and the Ang II abdominal versus thoracic aorta (736 genes), suggesting that ATF6 and the PERK ER stress pathways play more significant roles than the IRE1 pathway in promoting Ang II-induced AAA gene upregulation in ApoE-KO mice. In addition, in **Figure 6F**, the results showed that out of 11 trained immunity genes upregulated in Ang II+HFD AAA, ATF6-KO downregulated four, PERK-KO downregulated one, and IRE1-KO downregulated two. Out of 12 trained immunity genes upregulated in Ang II+HFD TAA, ATF6-KO downregulated eight, PERK-KO downregulated zero, and IRE1-KO downregulated one. These results suggest that the ER stress regulators ATF6, PERK, and IRE1 transcriptomes promote trained immunity.

Our previous reports showed that the antioxidant transcription factor NRF2 inhibits proinflammatory gene upregulation in peripheral blood mononuclear cells from patients with chronic kidney disease (66) and innate immune activation of vascular endothelial cells (67). As shown in **Figure 7A**, the upregulation of 466 genes upregulated in Ang II+HFD aortas was found in the Nrf2-KO dataset, suggesting that NRF2 inhibits Ang II-induced, Ang II+HFD-induced AA gene upregulation. In addition, the downregulations of 42 genes were found in the ROS-generating enzyme NOX2-KO dataset, suggesting that NOX2 promotes Ang II-induced AAA gene upregulation in ApoE-KO but not on a scale as large as that of NRF2 suppression. Moreover, downregulations of 3 genes were found in the PRKCD (ROS regulator located in ER)-KO dataset, suggesting a role for PRKCD in facilitating the progression of AAA. In addition, out of 11 trained immunity genes upregulated in Ang II+HFD AAA, NRF2-KO upregulated seven, NOX2-KO downregulated seven, and PRKCD-KO downregulated zero. Out of 12 trained immunity genes upregulated in Ang II+HFD TAA, NRF2-KO upregulated eight, NOX2-KO downregulated eight, and PRKCD-KO downregulated one. These results suggest that ROS promote trained immunity gene upregulation.

Trained immunity-promoting master gene SET domain containing 7 histone lysine methyltransferase (SET7) plays significant roles in promoting upregulation of secretomic genes in aortic and vascular cells. We found that the downregulation of 142 genes was found in the SET7-KD dataset, indicating that SET7 promotes trained immunity and the pathogenesis of Ang II-HFD AAA.

We then performed a Venn diagram analysis followed by pathway analysis to further determine the roles of the ATF6-upregulated transcriptome and the PERK-upregulated transcriptome in Ang II+HFD AAA and TAA in ApoE-KO mice. As shown in **Figure 7B**, the 222 ATF6-promoted genes upregulated in Ang II abdominal aorta had the top 20 pathways, including matrisome, inflammatory response, leukocyte activation, IL-10 signaling, neutrophil degranulation, defense response, response to external stimulus, cytokine production, TNFα superfamily cytokine production, and other 11 pathways. The 221 ATF6-promoted genes upregulated in the Ang II thoracic aorta had the top 20 pathways, including regulation of cell activation, cell activation, inflammatory response, cytokine production, matrisome, immune effector process, adaptive immune, and other 13 pathways (**Figure 7C**). The 270 ATF6-promoted genes unregulated in the Ang II abdominal versus thoracic aorta had the top 20 pathways, including synaptic signaling, neuron projection, neurotransmitter transport, regulation of membrane potential, and other 16 pathways (**Figure 7D**). As shown in **Figures 7E–G**, 19 PERK-promoted genes upregulated in the Ang II abdominal aorta had seven pathways, such as TNF signaling, blood vessel development, inflammatory, regulation of leukocyte activation, matrisome, secretion, and neutrophil degranulation pathways; 15 PERK-promoted genes upregulated in Ang II thoracic aorta had five pathways such as angiogenesis, neutrophil degranulation, skeletal development, neuron projection, and matrisome pathways; and 21 PERK-promoted genes upregulated in the Ang II abdominal versus thoracic aorta had four pathways, including neuron projection, brain development, protein localization to membrane, and behavior pathways. Taken together, our results have demonstrated that 1) ATF6 and PERK ER stress pathways play more significant roles than the IRE1 pathway in promoting Ang II-induced AAA gene upregulation as well as trained immunity upregulation in ApoE-KO mice; 2) antioxidant transcription factor NRF2 inhibits but ROS generating enzyme NOX2 promotes Ang II-induced AAA gene upregulation as well as trained immunity upregulation in ApoE-KO mice; 3) trained immunity master gene SET7 promotes trained immunity and pathogenesis of Ang II-HFD AAA; and 4) ER stress regulators ATF6, PERK play more important roles than IRE1 and synergistically upregulate the transcriptomes of Ang II+HFD AAA and TAA.

## Discussion

For the past 20 years, significant progress has been made to demonstrate the role of the Ang II system in promoting hypertension and vascular inflammatory response, especially in the Ang II-induced AAA mouse model (6). However, a key question remains to be addressed, underlying what kind of inflammation amplifying mechanisms does the Ang II-induced aneurysm prefer to happen in a specific area of the aorta, abdominal aorta, but not the whole aorta? Trained immunity describes the persistent hyperresponsive phenotypes in immune cells, including vascular endothelial cells (10, 13, 67) and vascular smooth muscle cells (11, 14), as we reported, which are acquired *via* metabolic reprogramming and amplify the innate immune response after brief stimulation (7, 68, 69). Our finding for the first time illustrates that Ang II, a well-documented risk factor for systemic hypertension and remodeling, pulmonary arterial hypertension, heart failure, myocardial infarction, aortic aneurysm, and inflammation (70), has the ability in an HFD-fed ApoE-KO background to bypass HFD-induced metabolic reprogramming and amplify inflammatory responses in the abdominal aorta but not the thoracic aorta. Targeting Angiotensin II levels by using Angiotensin-converting enzyme inhibitors (ACEIs) is the most commonly used medication for the treatment of cardiovascular diseases, including but not limited to heart failure, acute coronary syndrome, and hypertension (71, 72). Since hypercholesterolemia patients have a trained immunity phenotype, as we reported (8, 73, 74), which accelerates vascular inflammation and atherosclerosis (44, 45); and hypercholesterolemia stimulates angiotensin peptide synthesis (75), our finding provides a novel insight that targeting Ang II levels by using ACEIs may inhibit trained immunity in patients with hypercholesterolemia, atherosclerosis, heart failure, myocardial infarction, and aortic aneurysms.

Recent single-cell RNA-Seq data showed that VSMCs can be transdifferentiated into six cell phenotypes (76), endothelial-mesenchymal transition has also been found in cardiovascular diseases (77); and trans-differentiation of endothelial cells to smooth muscle cells plays an important role in vascular remodeling (78). It has been reported that many cell types, including VSMCs, endothelial cells, neutrophils, monocyte/macrophages, lymphocytes, adipocytes, mast cells, and platelets, contribute to the pathogenesis of aortic aneurysms (3). However, an important question remains whether cell transdifferentiation takes place in the pathogenesis of AAA (55). By examining the transcriptomic changes of the cell markers (8065 cell markers) of whole 79 cell types, we found that: *1)* the markers of immune cells were significantly upregulated in both the abdominal aorta and the thoracic aorta of Ang II-induced AAA; *2)* the markers of neuron cells and glial cells were upregulated by Ang II stimulation, indicating the roles of the newly established nervous system in the pathogenesis of AAA; and *3)* compared the abdominal aorta with the thoracic aorta, the markers of squamous epithelial cells were only significantly upregulated in the abdominal aorta but not in the thoracic aorta. Our new findings on aortic aneurysm-related trans-differentiation may be partially correlated with previous reports that VSMCs in the ascending aorta are derived from neural crest stem cells and progenitor cells in the second heart field, while VSMCs in the descending aorta are derived from somites (20, 79).

To summarize our findings here, we propose a new working model. *First*, we identified that Angiotensin II stimulation bypasses HFD-induced metabolic reprogramming and induces strong inflammatory responses in the abdominal aorta but not in the thoracic aorta of ApoE-KO mice, which provided a novel insight to explain why aneurysms mostly happen in the abdominal aorta. *Second*, as shown in **Figure 7H**, we found that most of the upregulated genes in the abdominal and thoracic aorta are not overlapped (75.8% in the abdominal aorta, 72.6% in the thoracic aorta) in response to Ang II-HFD stimulation, which indicates that different sections of the aorta have different transcriptomic signatures in pathological conditions. Of note, the vascular cells under ER stress may not be able to maintain stiffness efficiently, which will decrease vasoconstriction, impair relaxation and promote the development of aneurysm (80, 81). The major findings in the paper on 10 thoracic aneurysm models (80)indicated that intramural cells in the ascending aorta of mice prone to aneurysms are unable to maintain or restore the intrinsic circumferential material stiffness, which may render the wall biomechanically vulnerable to continued dilatation and possible rupture. Of note, 3 out of 8 gene mutation-based thoracic aneurysm mouse models listed were fibronectin-related. Along the same line, our new data (**Supplementary Figure 1**) illustrated the extracellular matrix components were partially differentially expressed in abdominal aorta or thoracic aorta, which further suggested the different cell stiffness in two aortic segments. For example, fibronectin 1 (FN1) was significantly upregulated only in the thoracic aorta. In contrast, the matrix metalloproteinase family gene including mmp13 and mmp14 were only upregulated in abdominal aorta.” *Third*, we demonstrated that the upregulated genes in the aortas of patients with abdominal aortic aneurysm, Ang II abdominal aorta, and thoracic aorta are partially overlapped with the interactomes of ER stress regulators ATF6, PERK, and IRE1, HPA-ER localization protein genes, and KEGG ER stress signaling genes. *Finally*, we found that the ATF6 and PERK ER stress pathways play more significant roles than the IRE1 pathway in promoting Ang II-ApoE KO AAA gene upregulation as well as trained immunity upregulation, whereas antioxidant NRF2 inhibited them. Combining them together, our unprecedented ER-focused transcriptomic analyses have provided novel insights on the roles of ER as an immune organelle in sensing various DAMPs and initiating ER stress that triggers Ang II-accelerated trained immunity and differs susceptibilities of thoracic and abdominal aortas to diseases.

## Data availability statement

The data presented in the study are deposited in the NIH-NCBI, BioProject accession number: PRJNA1004189.

## Ethics statement

The animal study was approved by IACUC of Temple University School of Medicine. The study was conducted in accordance with the local legislation and institutional requirements.

## Author contributions

YL: Data curation, Formal Analysis, Methodology, Writing – original draft. YSu: Formal Analysis, Writing – review & editing. FS: Writing – review & editing. YSh: Software, Writing – review & editing. KX: Writing – review & editing. XJ: Writing – review & editing. SW: Writing – review & editing. JY: Writing – review & editing. NS: Writing – review & editing. LY: Writing – review & editing. XS: Writing – review & editing. HZ: Formal Analysis, Methodology, Software, Writing – review & editing. HW: Writing – review & editing. XY: Conceptualization, Writing – review & editing.
