# Supplementary material for: ER stress mediates Angiotensin II-augmented innate immunity memory and facilitates distinct susceptibilities of thoracic from abdominal aorta to aneurysm development
Source: Front Immunol. 2023 Sep 4;14:1268916. doi: 10.3389/fimmu.2023.1268916 (PMC10507336; doi:10.3389/fimmu.2023.1268916)
Supplement: Supplementary file 1 [file Presentation_1.pptx]

## Slide 1
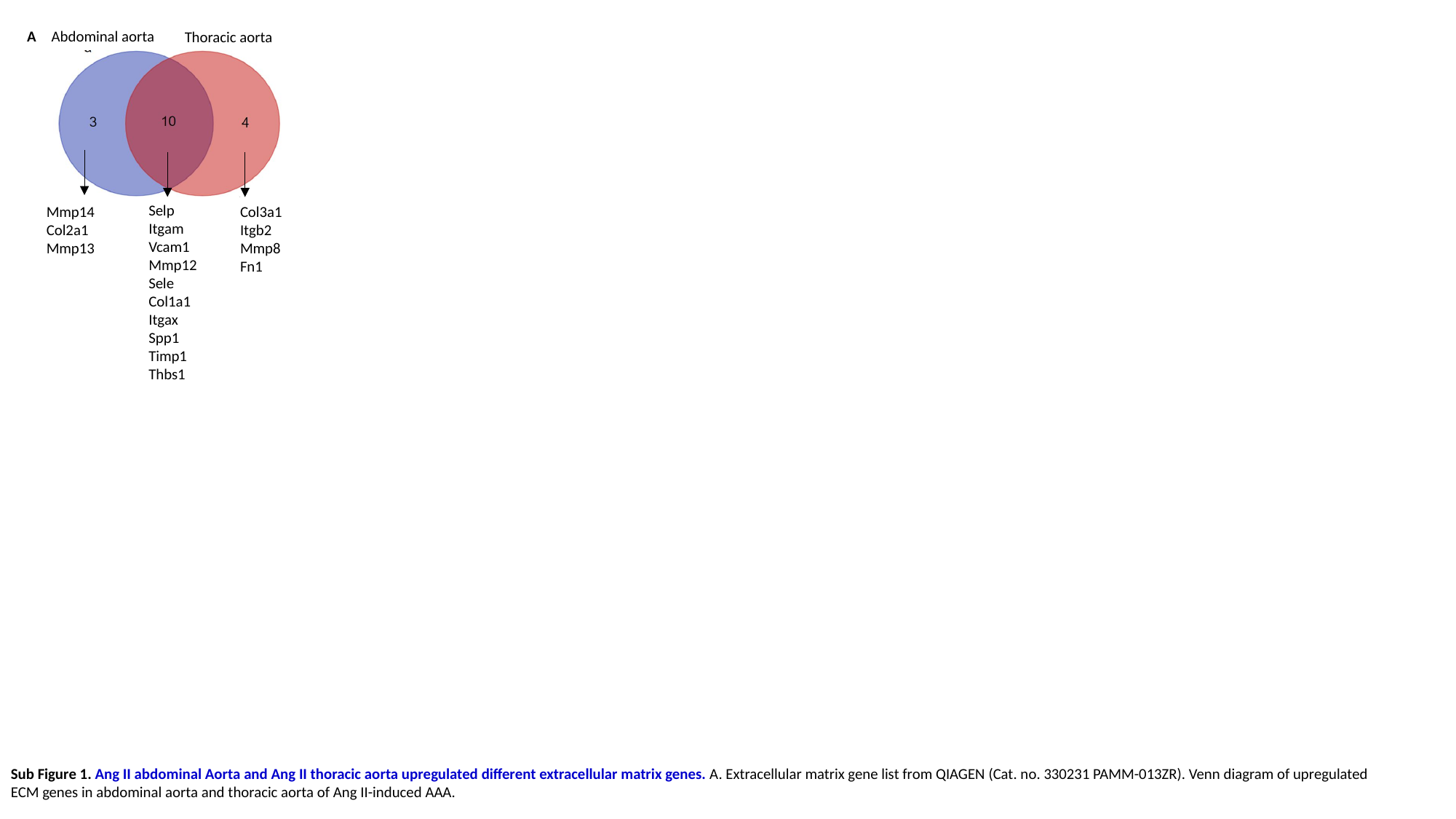

A
Abdominal aorta
Thoracic aorta
Selp Itgam Vcam1 Mmp12 Sele Col1a1 Itgax Spp1 Timp1 Thbs1
Mmp14 Col2a1 Mmp13
Col3a1 Itgb2 Mmp8 Fn1
Sub Figure 1. Ang II abdominal Aorta and Ang II thoracic aorta upregulated different extracellular matrix genes. A. Extracellular matrix gene list from QIAGEN (Cat. no. 330231 PAMM-013ZR). Venn diagram of upregulated ECM genes in abdominal aorta and thoracic aorta of Ang II-induced AAA.
